# Complications and clinical factors associated with pediatric percutaneous endoscopic gastrostomy in a Colombian cohort

**DOI:** 10.3389/fped.2025.1623355

**Published:** 2025-08-11

**Authors:** Carlos Augusto Cuadros Mendoza, Mario Javier Rosero Portilla, Verónica Pico Quintero, Edgar Fabián Manrique-Hernández, Alexandra Hurtado-Ortiz, Maricel Licht-Ardila, Alejandra Mendoza-Monsalve

**Affiliations:** ^1^ Department of Gastroenterology, Fundación Cardiovascular de Colombia, Piedecuesta, Colombia; ^2^ Postgraduate Department in Pediatric Critical Care, Universidad de Santander, Bucaramanga, Colombia; ^3^ Epidemiology Department, Fundación Cardiovascular de Colombia, Piedecuesta, Colombia

**Keywords:** endoscopy, gastrostomy, pediatrics, postoperative complications, enteral nutrition

## Abstract

**Introduction:**

Malnutrition significantly impairs both physical and cognitive function, increasing the risk of morbidity and mortality, especially in patients lacking a safe and effective route for enteral nutrition. Percutaneous endoscopic gastrostomy offers a minimally invasive solution for long-term enteral nutrition in pediatric patients, with a lower risk of perioperative complications compared to surgical alternatives.

**Objective:**

To evaluate the frequency, timing, and clinical factors associated with postoperative complications following pediatric percutaneous endoscopic gastrostomy.

**Methods:**

A retrospective analytical cohort study was conducted, including pediatric patients (≤18 years) who underwent Percutaneous endoscopic gastrostomy placement between January 2018 and December 2024. Bivariate analyses and Kaplan–Meier survival curves were used to assess the frequency of complications and complication-free survival time.

**Results:**

A total of 86 pediatric patients underwent Percutaneous endoscopic gastrostomy during the study period, of whom 12 (14%) experienced major postoperative complications. The median age was 4.53 years (interquartile range: 1.56–9.46 years). The most frequent major complication was Buried Bumper Syndrome, observed in 9 patients (10.47%). Minor complications included mild peristomal infection (8.14%) and feeding intolerance (5.81%). A complication-free survival of 96.73% (95% CI: 87.26–99.19) by day 12 and 69.35% (95% CI: 45.33–84.43) by day 40 was determined.

**Discussion:**

This study underscores the importance of systematic nutritional assessment and optimized post-operative care to reduce complications following PEG in pediatric patients. The high incidence of Buried Bumper Syndrome calls for more stringent follow-up protocols, especially in resource-limited settings. Close monitoring during the early post-operative period can prevent complications.

## Introduction

Malnutrition is defined by alterations in body composition that negatively impact both physical and cognitive function, increasing morbidity and mortality, particularly in patients with underlying medical conditions (1). Its consequences include sarcopenia, immunosuppression, delayed wound healing, and a higher susceptibility to infections (2). The absence of a safe and effective route for enteral nutrition may exacerbate malnutrition, leading to further complications such as bronchial microaspirations and increased dependence on parenteral nutrition (3, 4).

Establishing adequate enteral access is therefore critical for meeting nutritional requirements, especially during periods of metabolic stress (5). The choice of access route depends on the underlying pathology and expected duration of nutritional support (6). While nasogastric tubes are typically used for short-term feeding, gastrostomy is recommended when enteral nutrition is anticipated to last longer than 4–6 weeks (7). In pediatric patients, percutaneous endoscopic gastrostomy (PEG) has become the preferred method, offering a minimally invasive alternative to surgical approaches, with a lower risk of perioperative complications and faster recovery (4).

PEG is particularly advantageous in children with complex comorbidities—such as congenital heart disease or neurological disorders—where timely and sustained nutritional support can improve clinical stability and quality of life (7). Nonetheless, the indications for PEG vary by clinical context and population, encompassing conditions like neurogenic dysphagia, congenital oropharyngeal anomalies, and oncological diseases (8). These differences underscore the importance of a context-specific evaluation when determining candidacy for the procedure (9).

In addition to appropriate patient selection, monitoring and understanding both early and late complications are essential for optimizing care and minimizing risks (10). Complications such as infections, tube dislodgement, and respiratory issues must be identified and managed proactively to improve outcomes and guide preventive strategies (9, 11). In this context, the present study aims to evaluate the frequency, timing, and clinical factors associated with postoperative complications following pediatric PEG.

## Methods

**Figure 1:**
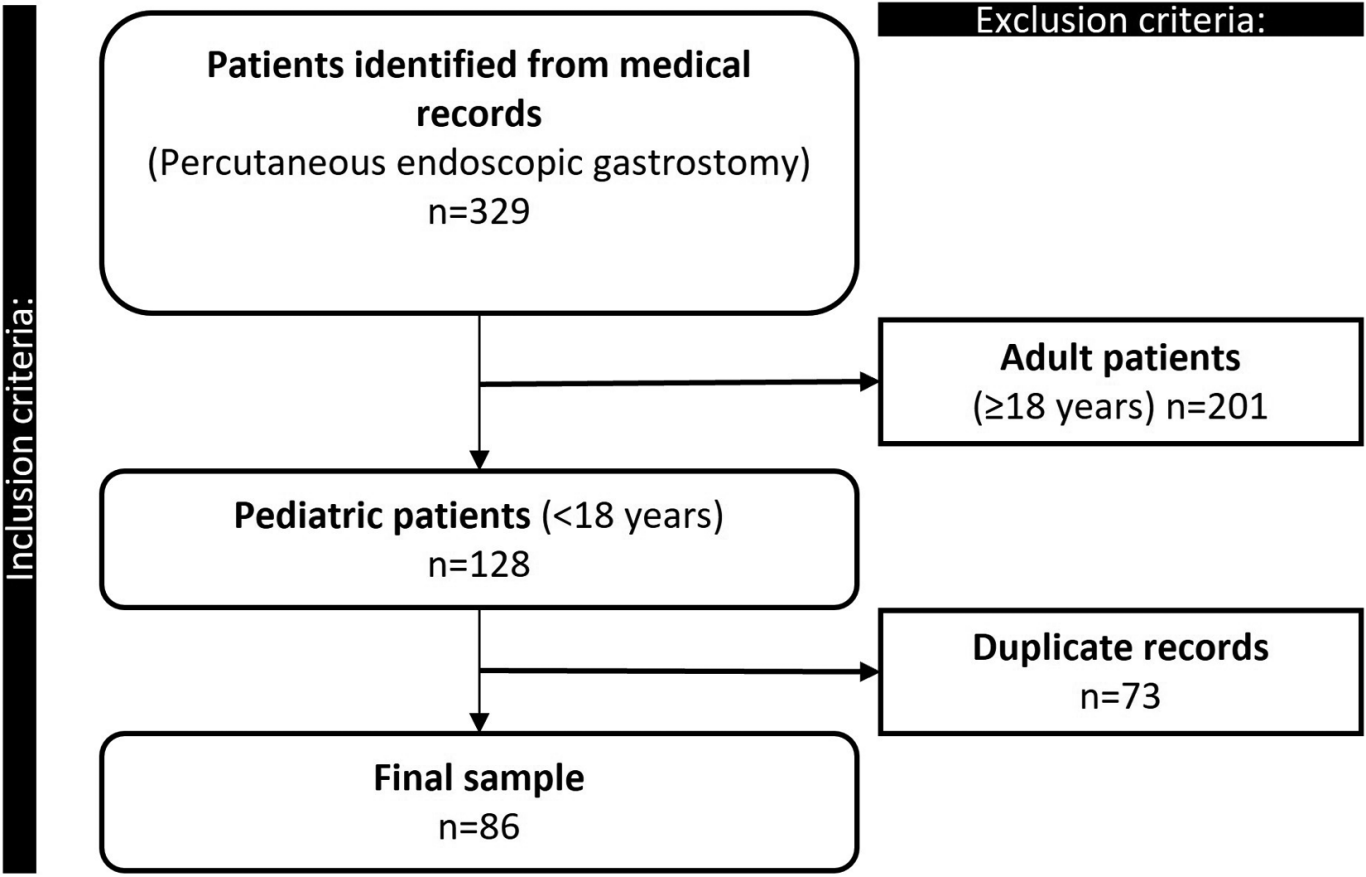
Flowchart summarizing the selection process of pediatric patients undergoing endoscopic gastrostomy.

A retrospective analytical cohort study was conducted, including all pediatric patients (≤18 years) who underwent PEG at a high-complexity hospital in northeastern Colombia between 01 January 2018 and 08 December 2024. Data were extracted from the institutional electronic medical records and correspond to the hospitalization during which the gastrostomy procedure was performed (Figure 1). All researchers involved in data collection and processing received training to minimize potential errors and ensure data reliability.

The following variables were collected: Sociodemographic variables: sex, age, weight and height at the time of the procedure and discharge. Clinical variables: nutritional status at the time of the procedure, and pre-existing comorbidities, including cystic fibrosis, chronic kidney disease, non-progressive or progressive encephalopathy, neuromuscular or neurometabolic diseases, congenital heart disease, sequelae of traumatic brain injury or cerebrovascular events, gastroesophageal reflux disease, and recent extracorporeal membrane oxygenation (ECMO) use within the past 3 months. Pre-procedure laboratory values were also recorded: albumin level, hemoglobin, and international normalized ratio (INR).

Procedure-related variables included the indication for PEG—such as severe oral hypersensitivity, refractory dysphagia, refractory failure to thrive, eating behavior disorders, and craniofacial malformations—date of the procedure, occurrence of intraoperative complications, and type of post-procedural enteral nutrition (normocaloric vs. hypercaloric; elemental vs. polymeric). Additional data included changes in the gastrostomy tube (e.g., removal with internal bumper), reasons for removal (routine replacement, accidental displacement, or complications), the need for laparoscopic gastrostomy, and length of hospital stay (from admission for the procedure to discharge).

Outcomes: The primary outcome was the occurrence of postoperative complications, classified as major or minor, according to the Dindo-Clavien classification (12). *Major complications* included Buried Bumper Syndrome (BBS), peritonitis, significant gastrointestinal bleeding (requiring hemostatic intervention, transfusion, or endoscopic/surgical therapy), severe peristomal infection, soft tissue sepsis (complete blood count and acute-phase reactants), gastrocutaneous or enterocutaneous fistula (contrast-enhanced digestive tract radiography with water-soluble contrast and digestive endoscopy), peristomal abscess, fascial necrosis (surgical confirmation is necessary), visceral perforation, tension pneumoperitoneum (plain abdominal x-ray), and death. *Minor complications* comprised mild peristomal infections, leakage, ostomy dilation, granuloma formation, accidental tube removal, tube displacement without evidence of peritonitis or pneumoperitoneum, pain unresponsive to The World Health Organization step-1 analgesics, and feeding intolerance.

The procedures were performed by a pediatric surgeon with over 10 years of experience in minimally invasive surgery, who conducted 3.3% of the cases. A recently graduated pediatric surgeon performed an additional 3.3% of the procedures. The majority of the procedures (73.3%) were carried out by a pediatric gastroenterologist with more than 10 years of experience in endoscopic techniques. Additionally, a pediatric gastroenterologist-nutritionist, also with extensive experience, was responsible for 22% of the procedures. All professionals had significant prior experience in performing PEG and adhered to standardized institutional protocols, ensuring the safety and consistency of the procedure throughout the study cohort.

### Surgical technique

Following the identification of the indication for PEG, a consultation is requested with the pediatric gastroenterology service to confirm the indication and determine the optimal timing for the procedure, based on the patient's nutritional status and clinical stability. The procedure is scheduled electively in the operating room, following informed consent. Sedation is administered, and depending on the clinical context, the airway is secured via endotracheal intubation.

In the standard technique, the pediatric gastroenterologist performs an upper endoscopy to identify the optimal site for insertion under aseptic conditions. A long guidewire is introduced endoluminally into the stomach and retrieved percutaneously using the pull-through technique. An age- and size-appropriate gastrostomy tube is inserted as follows: 14 Fr for small children under 5 years of age, 20 Fr for older children aged 5–12 years, and 22–24 Fr for adolescents over 11 years of age. The tube is secured with an internal retention dome, and proper intragastric positioning is confirmed endoscopically. The tube is kept closed for 24 h with monitoring of clinical signs; feeding is initiated thereafter according to clinical status.

### Follow-up protocol

Patients are routinely monitored in the pediatric ward or intensive care unit until full enteral feeding volume is achieved. Upon discharge, a follow-up visit is scheduled at 1 month, during which the internal bolster gastrostomy tube is electively replaced with a balloon gastrostomy tube. This replacement is performed endoscopically in a planned manner.

### Statistical analysis

Categorical variables were reported as absolute frequencies and percentages, while continuous variables were tested for normality using the Shapiro–Wilk test. Variables with non-normal distribution were expressed as medians with interquartile ranges (IQR). Bivariate analyses were conducted to explore associations between independent variables and the occurrence of major complications. Categorical variables were compared using the chi-square or Fisher's exact test, and non-normally distributed continuous variables were compared using the Mann–Whitney *U* test. Survival analysis was performed using Kaplan–Meier curves to estimate complication-free survival, with comparisons made using the log-rank test. A two-tailed *p*-value <0.05 was considered statistically significant. All analyses were performed using STATA version 16 (StataCorp, USA).

## Results

Patients were classified into two groups based on the presence or absence of postoperative complications (Figure 1). A total of 86 pediatric patients were included, of whom 51 (59.30%) were male and 35 (40.70%) were female. In the group without complications, 46 patients (62.16%) were male, whereas in the group with complications, 5 (41.67%) were male. The overall median age was 4.53 years, with similar values observed across both groups (no complications: 4.53 and complications: 4.39). Weight and height at the time of the procedure were also comparable, with a median weight of 12.60 kg and a median height of 94.50 cm. At hospital discharge, the median weight and height remained similar between groups, at 12.45 kg and 93.00 cm, respectively (Table 1).

**Table 1:** Sociodemographic characteristics of pediatric patients according to the presence of major complications.

Variable	Category	No complication *n* = 73 (%)	Complication *n* = 12 (%)	Total *n* = 86 (%)	*p*-value
Sex	Female	28 (37.84)	7 (58.33)	35 (40.70)	0.180
	Male	46 (62.16)	5 (41.67)	51 (59.30)	
Age (years)	Median (IQR)	4.53 (1.56–9.46)	4.39 (1.64–8.15)	4.53 (1.56–9.46)	0.654
Weight at procedure (kg)	Median (IQR)	13 (8–27)	11.15 (8–14.95)	12.6 (8–22.50)	0.307
Height at the procedure (cm)	Median (IQR)	96 (75–143)	91 (79.55–100)	94.5 (76–123)	0.529
Discharge weight (kg)	Median (IQR)	13 (8.30–23.90)	11.93 (9.95–16.15)	12.45 (8.30–22.50)	0.638
Discharge Height (cm)	Median (IQR)	93.50 (75–130)	90 (79.5–98.50)	93 (75.20–123)	0.586

IQR, interquartile range.

The analysis of clinical history revealed no statistically significant differences in most of the variables assessed. Severe acute malnutrition and exacerbated chronic malnutrition were more frequent among patients who developed complications (16.67% and 25.00%, respectively). Similarly, no significant associations were found with encephalopathy, neuromuscular diseases, congenital heart disease, chronic lung disease, or sequelae of cerebrovascular events. Swallowing disorders were present in 94.19% of patients. Although a higher proportion of patients with complications received a hypercaloric diet compared to those without complications (33.33% vs. 13.70%, *p* = 0.089). Additionally, pre-procedure levels of albumin, hemoglobin, and INR were not associated with the development of complications (Table 2).

**Table 2:** Clinical characteristics of pediatric patients according to the presence of major complications.

Variable	Category	No complication *n* = 73 (%)	Complication *n* = 12 (%)	Total *n* = 86 (%)	*p*-value
Medical history					
Malnutrition	Mild-to-moderate	3 (4.05)	0 (0)	3 (3.49)	0.478
	Severe acute	4 (5.41)	2 (16.67)	6 (6.98)	0.155
	Exacerbated chronic	6 (8.11)	3 (25.00)	9 (10.47)	0.076
	Chronic in recovery	1 (1.35)	0 (0)	1 (1.16)	0.685
Cystic fibrosis	No	74 (100)	12 (100)	86 (100)	
Encephalopathy	Non-progressive	18 (24.32)	3 (25.00)	21 (24.42)	0.96
	Progressive	10 (13.51)	1 (8.33)	11 (12.79)	0.618
Neuromuscular disease	Yes	1 (1.35)	0 (0)	1 (1.16)	0.685
Malignancy	Yes	7 (9.46)	0 (0)	7 (8.14)	0.266
Orofacial malformations	Yes	1 (1.35)	0 (0)	1 (1.16)	0.685
Chronic kidney disease	Yes	1 (1.35)	0 (0)	1 (1.16)	0.685
Sequelae of hypoxic-ischemic encephalopathies	Yes	10 (13.51)	2 (16.67)	12 (13.95)	0.77
Chronic lung disease	Yes	6 (8.11)	0 (0)	6 (6.98)	0.306
Sequelae of cerebrovascular events	Yes	11 (14.86)	1 (8.33)	12 (13.95)	0.545
Congenital heart disease	Yes	9 (12.16)	2 (16.67)	11 (12.79)	0.665
Neurometabolic disease	Yes	8 (10.81)	1 (8.33)	9 (10.47)	0.795
Sequelae of traumatic brain injury	Yes	5 (6.76)	0 (0)	5 (5.81)	0.353
Muscular dystrophy	No	74 (100)	12 (100)	86 (100)	
Gastroesophageal reflux disease	Yes	2 (2.7)	1 (8.33)	3 (3.49)	0.324
Requires ECMO in the last 3 months	Yes	4 (5.41)	1 (8.33)	5 (5.81)	0.688
Failure to thrive	Yes	1 (1.35)	0 (0)	1 (1.16)	0.685
Swallowing disorder	Yes	70 (94.59)	11 (91.67)	81 (94.19)	0.688
Eating behavior disorder	No	74 (100)	12 (100)	86 (100)	
Oral hypersensitivity disorder	Yes	2 (2.70)	0 (0)	2 (2.33)	0.564
Access for medications	Yes	2 (2.70)	1 (8.33)	3 (3.49)	0.324
Cranio-orofacial malformations	Yes	1 (1.35)	0 (0)	1 (1.16)	0.685
Concomitant surgeries	Yes	2 (2.70)	1 (8.33)	3 (3.49)	0.324
Complications during the procedure	Yes	2 (2.70)	0 (0)	2 (2.33)	0.564
Nutrition after gastrostomy	Hypercaloric	10 (13.70)	4 (33.33)	14 (16.47)	0.089
	Normocaloric	63 (86.30)	8 (66.67)	71 (83.53)	
Albumin (g/dl)^a^	Median (IQR)	3.59 (3.07–3.96)	4.01 (3.37–4.26)	3.66 (3.07–4.26)	0.536
Pre-procedure HB (g/dl)	Median (IQR)	11.2 (9.98–12.8)	11.55 (9.335–12.65)	11.20 (9.97–12.8)	0.677
INR	Median (IQR)	1.04 (0.99–1.14)	1.09 (0.97–1.4)	1.05 (0.98–1.16)	0.403

^a^ IQR, interquartile range; Chronic lung disease = bronchopulmonary dysplasia; HB, hemoglobin; INR, international normalized ratio; ECMO, extracorporeal membrane oxygenation.

A total of 37 complications, 23 minor identified in 20 patients and 14 major in 12 patients, indicating that some individuals experienced more than one event. Major complications were observed in 16.30% of cases, with BBS being the most frequent (10.47%), followed by severe peristomal infection (3.49%), gastrostomy-related sepsis (1.16%), and gastrocutaneous fistula (1.16%). Minor complications were more prevalent and included mild peristomal infection (8.14%), feeding intolerance (5.81%), granuloma formation (3.49%) and accidental tube removal (3.49%) (Table 3).

**Table 3:** Frequency and types of complications in pediatric patients undergoing endoscopic gastrostomy.

Complication type	Complication name	*n* (%)
Major complications	Buried bumper syndrome	9 (10.47)
	Severe peristomal infection	3 (3.49)
	Sepsis (gastrostomy-related)	1 (1.16)
	Gastrocutaneous fistula	1 (1.16)
Minor complications	Mild peristomal infection	7 (8.14)
	Gastric content leakage	2 (2.33)
	Ostomy dilation	2 (2.33)
	Granuloma formation	3 (3.49)
	Accidental tube removal	3 (3.49)
	Feeding intolerance	5 (5.81)
	Need for reintervention	1 (1.16)

**Figure 2:**
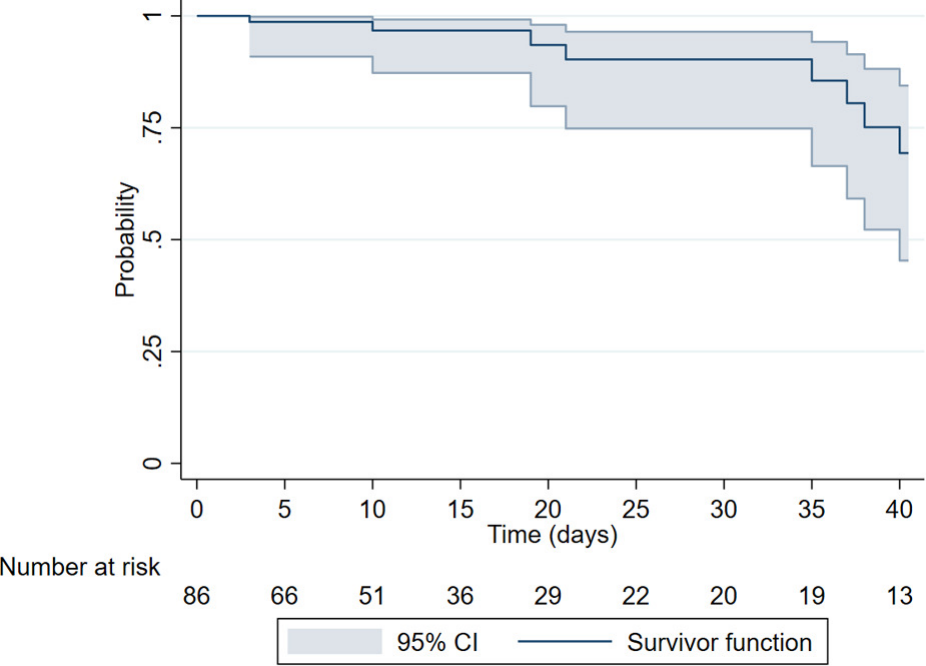
Kaplan–Meier curve of 40-day major complication-free survival following endoscopic gastrostomy.

Kaplan–Meier analysis revealed a progressive decline in major complication-free survival following the procedure. The probability of remaining free of major complications by day 12 was 96.73% [95% Confidence Interval (CI): 87.26–99.19], and by day 29, it was 90.28% (95% CI: 74.84–96.46). The most pronounced drop occurred after day 36, with the probability decreasing to 75.13% (95% CI: 52.24–88.17) by day 38 and to 69.35% (95% CI: 45.33–84.43) by the end of follow-up (day 40) (Figure 2).

**Figure 3:**
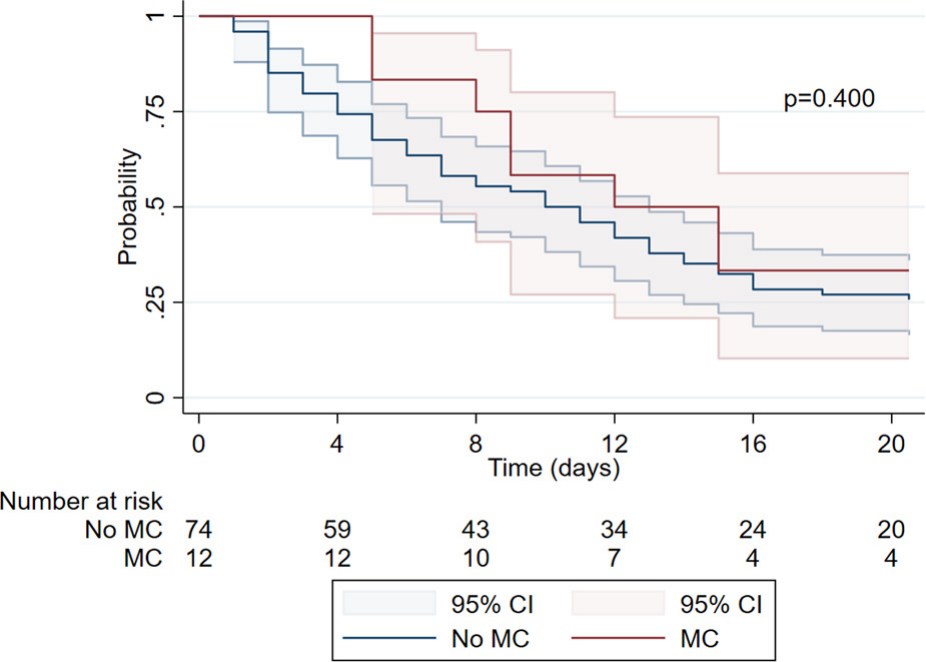
Kaplan–Meier analysis, length of stay post-gastrostomy. MC, major complications present; No MC, no major complications.

The analysis of hospital length of stay after gastrostomy demonstrated differences based on the presence of major complications. In patients without major complications, the probability of remaining hospitalized progressively decreased, with only 25.68% (95% CI: 16.40–35.98) still hospitalized by day 20, suggesting that 74.32% had been discharged by that point. In contrast, patients who experienced a major complication showed a more pronounced decline in the probability of remaining hospitalized, with only 33.33% (95% CI: 10.27–58.84) still inpatient by day 15 (*p* = 0.400) (Figure 3).

## Discussion

In this cohort of 86 pediatric patients who underwent PEG, the overall complication rate was 13.95%, which aligns with data reported in the pediatric literature. A similar complication rate of 14% was found in a prospective study involving 103 children, suggesting that the safety profile observed in our population is consistent with previous findings (13). The complication profile was heterogeneous, with minor complications such as mild peristomal infections and feeding intolerance being more frequent than major complications. Of particular note, the presence of severe acute malnutrition and exacerbated chronic malnutrition was more common among those who experienced complications, which, although not statistically significant in our sample, aligns with evidence linking malnutrition to impaired wound healing and infection risk (14). This highlights the need to systematically assess and optimize nutritional status before to PEG placement to reduce the likelihood of postoperative complications.

The occurrence of granulomas and feeding intolerance in our cohort reflects patterns similar to those described internationally; however, the incidence of granuloma formation was lower than in larger studies. This difference suggests that local factors, such as stoma care protocols, caregiver education, nutritional management, and tube selection, may play a critical role in reducing complication rates. Proper wound care and close postoperative monitoring could help prevent excessive tissue irritation and promote better stoma healing, ultimately leading to fewer granulomas and improved feeding tolerance in pediatric patients (15).

BBS emerged as the most frequent major complication. This finding is particularly relevant, as the incidence reported in the literature ranges from 1% to 8%, with some recent studies indicating an increase in specific populations (16, 17). Factors such as the rigidity and material of the gastrostomy tube, as well as the external fixation device's position, can significantly influence the development of BBS. A tight external fixator, improper positioning, and excessive compression between the internal and external bumpers are key etiological factors leading to this complication, as noted in several studies (16, 18). This association is of particular interest in Latin American settings, where variations in resource availability and follow-up protocols may influence both the rate and management of complications (19).

In our cohort, peristomal infections ranked second among major complications and were the most frequent minor complication. This finding is consistent with previous literature, which identifies peristomal infection as one of the most common complications following PEG, with reported rates ranging from 4% to 30% depending on prophylactic measures, postoperative care, and the experience of the clinical team (20). Severe infections, such as gastrostomy-related sepsis, highlight the critical importance of close monitoring and meticulous stoma care to prevent the progression of local infection to systemic complications (21). Factors such as malnutrition, immune compromise, suboptimal hygiene, and variability in follow-up protocols further increase this risk, particularly in pediatric populations (22). Moreover, the lack of standardized caregiver education and perioperative antibiotic prophylaxis can significantly influence infection rates, emphasizing the need for preventive strategies based on international guidelines adapted to local contexts (20, 22).

Our Kaplan–Meier analysis revealed a progressive decline in complication-free survival over the 40-day follow-up period, with the majority of complications occurring within the first few weeks after the procedure. This finding aligns with the international literature, which consistently reports that most complications following PEG placement in pediatric populations arise within the initial 2–4 postoperative weeks. This underscores the critical importance of intensive clinical monitoring during this early post-operative period to promptly identify and manage potential complications, thereby improving patient outcomes and minimizing long-term risks (15).

The analysis of post-PEG hospital stay duration revealed that patients without major complications were typically discharged earlier, demonstrating a reduced probability of prolonged hospitalization. In contrast, those who experienced major complications required longer stays, likely due to the need for more intensive and complex post-operative management. These findings align with those reported by Fox et al. (23), who observed a similar trend in their cohort. The extended hospital stays in patients with complications highlight the critical role of early detection and timely interventions in preventing the escalation of post-operative issues, ultimately contributing to more efficient resource utilization and improved patient outcomes.

This study provides valuable data that enrich the limited evidence available on PEG outcomes in middle-income countries, particularly in Latin America, where original studies on complications are scarce. However, its retrospective design limits the ability to establish causal relationships, and the relatively small sample size may affect the generalizability of the findings, especially regarding the analysis of major complications, which were infrequent. Despite these limitations, our results offer relevant insights into the safety profile and specific challenges faced by pediatric patients in the region, contributing important context to the global understanding of PEG outcomes.

## Conclusion

PEG in the pediatric population proved to be a safe procedure, with a low overall complication rate, predominantly involving minor events. However, the relatively high incidence of BBS observed in this cohort underscores the need to strengthen postoperative follow-up, particularly during the early weeks after the procedure.

## Data Availability

The original contributions presented in the study are included in the article/Supplementary Material, further inquiries can be directed to the corresponding author.
