# Correction: Complications and clinical factors associated with pediatric percutaneous endoscopic gastrostomy in a Colombian cohort

**DOI:** 10.3389/fped.2025.1701519

**Published:** 2025-09-18

**Authors:** Carlos Augusto Cuadros Mendoza, Mario Javier Rosero Portilla, Verónica Pico Quintero, Edgar Fabián Manrique-Hernández, Alexandra Hurtado-Ortiz, Maricel Licht-Ardila, Alejandra Mendoza-Monsalve

**Affiliations:** ^1^ Department of Gastroenterology, Fundación Cardiovascular de Colombia, Piedecuesta, Colombia; ^2^ Postgraduate Department in Pediatric Critical Care, Universidad de Santander, Bucaramanga, Colombia; ^3^ Epidemiology Department, Fundación Cardiovascular de Colombia, Piedecuesta, Colombia

**Keywords:** endoscopy, gastrostomy, pediatrics, postoperative complications, enteral nutrition

Affiliation 2 Postgraduate Department in Pediatric Critical Care, Universidad de Santander, Bucaramanga, Colombia was omitted for author Carlos Augusto Cuadros Mendoza. This affiliation has now been added for author Carlos Augusto Cuadros Mendoza.

Affiliation 3 Epidemiology Department, Fundación Cardiovascular de Colombia, Piedecuesta, Colombia was omitted for authors Alexandra Hurtado-Ortiz, Maricel Licht-Ardila and Alejandra Mendoza-Monsalve. This affiliation has now been added for authors Alexandra Hurtado-Ortiz, Maricel Licht-Ardila and Alejandra Mendoza-Monsalve.

Authors Alexandra Hurtado-Ortiz, Maricel Licht-Ardila and Alejandra Mendoza-Monsalve were erroneously assigned to affiliation 2 Postgraduate Department in Pediatric Critical Care, Universidad de Santander, Bucaramanga, Colombia. This affiliation has now been removed for authors Alexandra Hurtado-Ortiz, Maricel Licht-Ardila and Alejandra Mendoza-Monsalve.

The original version of this article has been updated.

